# Spatio-temporal evolution and influencing factors of China’s ICT service industry

**DOI:** 10.1038/s41598-023-34994-z

**Published:** 2023-06-15

**Authors:** Weixuan Chen, Shiwei Zhang, Dezhou Kong, Tong Zou, Yuxi Zhang, Ali Cheshmehzangi

**Affiliations:** 1 Department of Architecture and Built Environment, University of Nottingham, Nottingham, NG7 2RD UK; 2 Institute of Geographic Sciences and Natural Resources Research, Chinese Academy of Sciences, Beijing, 100101 China; 3 Department of Architecture and Built Environment, University of Nottingham, Ningbo, 315100 China; 4 Hiroshima University, Hiroshima, 739-8530 Japan; 5 Qingdao City University, Qingdao, 266106 China

**Keywords:** Mathematics and computing, Information technology, Scientific data

## Abstract

The ICT service industry has become a burgeoning industry at a high and stable speed. Their equitable distribution can improve national and global positive peace. This paper aimed to verify the characteristics of spatio-temporal evolution and its influencing factors in the ICT service industry. Based on the data from 31 Provinces in China from 2015 to 2019, this paper uses location quotient, spatial autocorrelation methods and spatial econometric analysis to explore the development characteristics, evolution and influencing factors of the ICT service industry, respectively. The main results are shown as follows: (1) China's ICT service industry is mainly concentrated in Beijing, Shanghai, Zhejiang, Tibet, and Guangdong, with a trend of specialisation development. They are not only distributed in cities with relatively superior overall development but also those with superior industrial and development carrier elements. Technological relevance, aggregation, and political difference might have an impact on promoting the emergence and development of these industries. (2) ICT service industry is characterised by stable and highly concentrated development. Numbers between three to five significant provinces and types with high-high (HH) and high-low (HL) clusters of local spatio-temporal association kept stable in the period. The HH was in eastern coastal areas, including Zhejiang, Shanghai, Jiangsu, and Shandong, and the HL was in Guangdong in 2015. There is a definite spatial correlation in spatial distribution with constant strengthening. (3) TUR, NDN, MIAT and the area were shown to have a significant role in promoting the ICT service industry, while NW, GDP and ICT Employment were shown to have a significant negative impact on this industry. Correspondingly, two strategies were put forward here: (1) accelerating the inter-provincial networking development of the ICT service industry, and (2) strengthening government policy guidance for the ICT service industry. These outcomes can not only provide a scientific basis and theoretical support for the distribution of strategies and resources for these industries at the theoretical level but also improve resource integration from the national perspective and the efficiency of resource use at the practical level.

## Introduction

Globally, we are undergoing a vast digital transformation accelerated by the novel capabilities and technologies brought on by major implications that derive due to the COVID-19 outbreak and the 4th Industrial Revolution. During this process, Information and Communication Technology (ICT) has become a substantial part of the research on the environmental-social-economic sustainability research and its application in daily life and business. In the preceding decades, ICT is constantly gaining momentum and the ongoing digital transformation of the economy is reshaping and redefining business models and industrial production. Massive reduction in computing and communications costs has spurred huge investments^1,2^ and engendered a substantial restructuring of the economy^3^. Computers, mobile communication devices, and the internet are integrated into everyday lives and are consequently changing how business is done, and markets work in manifold ways^4^. ICT has been at the heart of economic changes for more than a decade and has proven to be resilient during the recent economic crisis. The ICT industry contributes to technological progress, output, and productivity growth, widely recognised as an innovative stimulus that can enhance economic and social development at different levels^4–6^, both in well-developed and in less-developed countries. Reaping the benefits of ICT has a drastic effect on various socio-economic aspects of countries worldwide^7^. Their impacts can be examined directly through their contribution to output, employment, or productivity growth; or indirectly, as a source of technological change affecting other parts of the economy^8^, playing an important role in promoting economic development.

**Figure 1:**
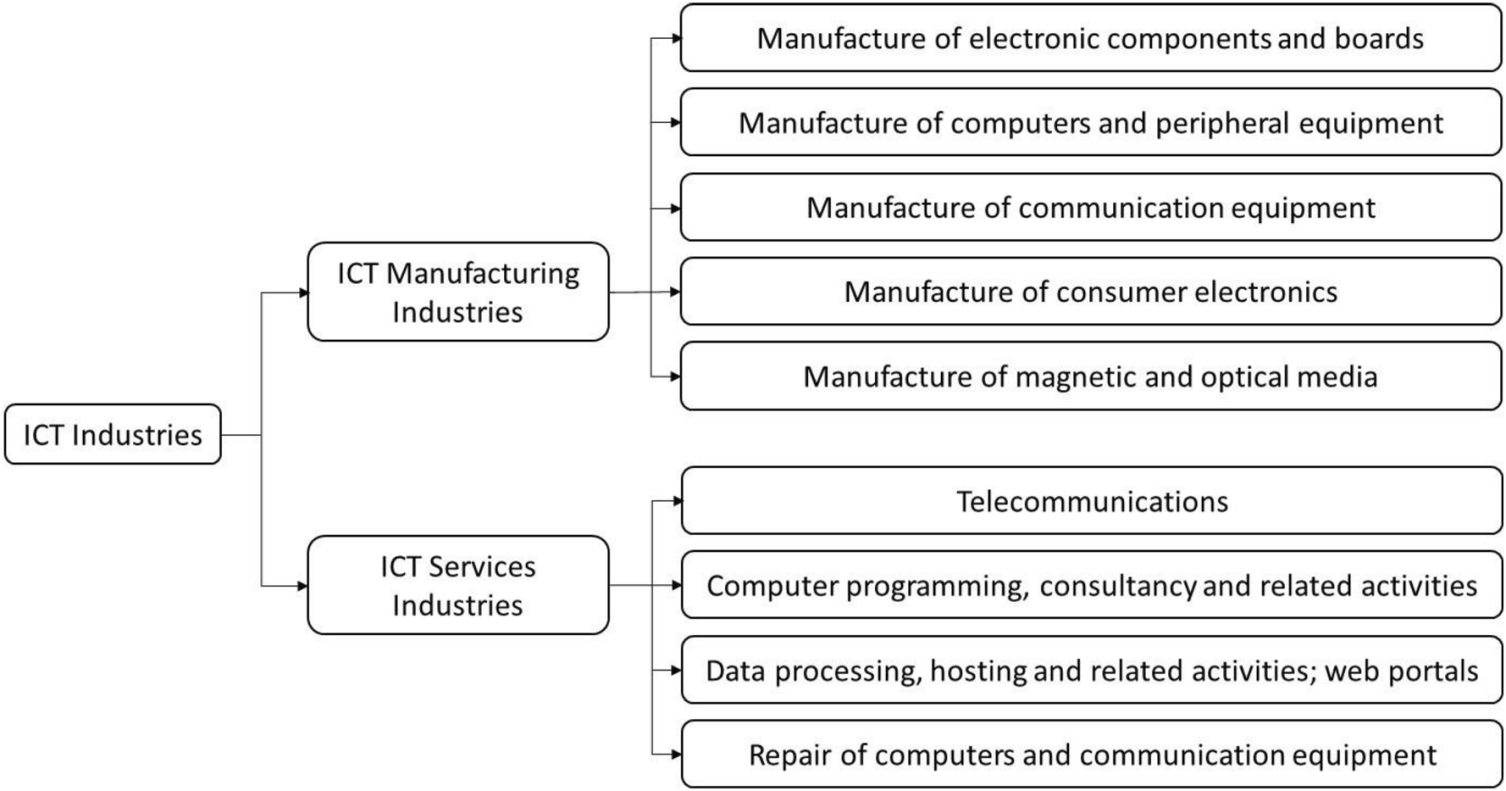
The classification of the ICT industry. Source: The authors’ edition based on OECD^9^ and World Bank^12^.

The term ICT is generally adopted to describe information management techniques, telecommunication technologies, devices and applications that enable the production, processing, grouping, retrieval, storage and transformation of information^9^. Its definitions vary differently over different periods, ranging from fixed-line telephones and computers to the Internet and mobile technologies. Nowadays, this is widely defined as a collection of capturing, transmitting, and displaying data and information electronically, divided into manufacturing and service industries^9^. Several studies have expanded the scope of the ICT umbrella term to account for applications that enable the generation and access to data, data collection, processing, storage, and transmission^10,11^. There is a general consensus that the term covers the three broad industries of telecommunications, computing, and broadcasting^12^. Figure 1 summarises the classification of the ICT industry. The ICT service industry was intended to enable the function of information processing and communication by electronic means (including telecommunications, computer programming, consultancy and related activities, data processing, and hosting and related activities), web portals, and repair of computers and communication equipment^9^. Since the ICT service industry is constantly gaining momentum as a significant driver of growth and general-purpose technologies that have a horizontal impact on all economic activities^13^, the investigation of their evolution and influencing factors within a given economy could shed light on the underlying mechanisms that define their profound and transformative effects.

**Figure 2:**
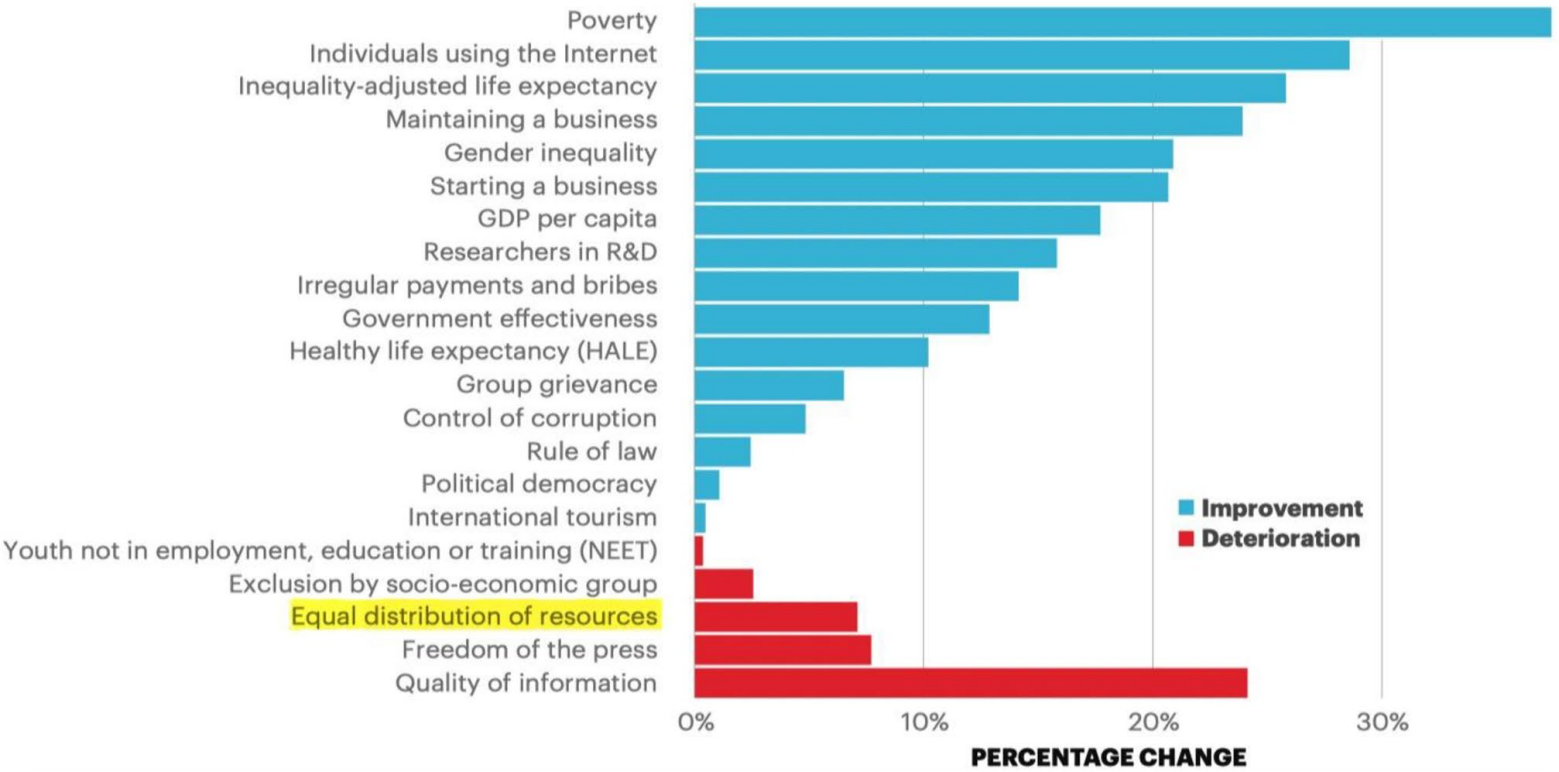
Percentage change in Positive Peace indicators, China, 2009–2019 Source: Institute for Economics and Peace^23^).

Many of these studies were conducted in European and North American countries^1,14–16^. In early studies, ICT research was frequently related to socio-economic development in developed countries. The pioneering study was “The role of the telephone in economic development”^17^. Later, the impact of computers on economic development in the US was analysed^18^. More recently, scholars have also considered such analysis in developing countries within their purview^19,20^. As the largest developing country, China can be a representative case of ICT studies to provide experience and reference for other developing countries, which is necessary and meaningful^21^. Compared with many cities in western countries, which often share a broadly similar economic and political history among them (e.g., free market or mixed economy, social-democratic systems, etc.), Chinese cities are very different, both economically and, above all, politically^22^. Over the past decade, China has experienced advances in economic, health, and physical infrastructure gauges, with a 7.4% improvement in its Positive Peace Index (i.e., the Positive Peace Index measures the level of Positive Peace. This provides a baseline measure of the effectiveness of a country’s capabilities to build and maintain peace. It also provides a measure for policymakers, researchers and corporations to use for effective intervention design, monitoring and evaluation) score^23^. ICT development plays a vital role in the progress, preservation, and appropriation of these activities^24,25^. Its improvements were posted in its sound business environment of positive peace along with a contrast of worse equitable distribution of resources (*note: The eight pillars of positive peace include: a well-functioning government; a sound business environment; an equitable distribution of resources; an acceptance of the rights of others; good relations with neighbours; free flow of information; a high level of human capital; and low levels of corruption*) (see Fig. 2). In this case, the distribution of the ICT service industry should be verified to improve the distribution of resources and to further achieve China’s and global positive peace.

**Figure 3:**
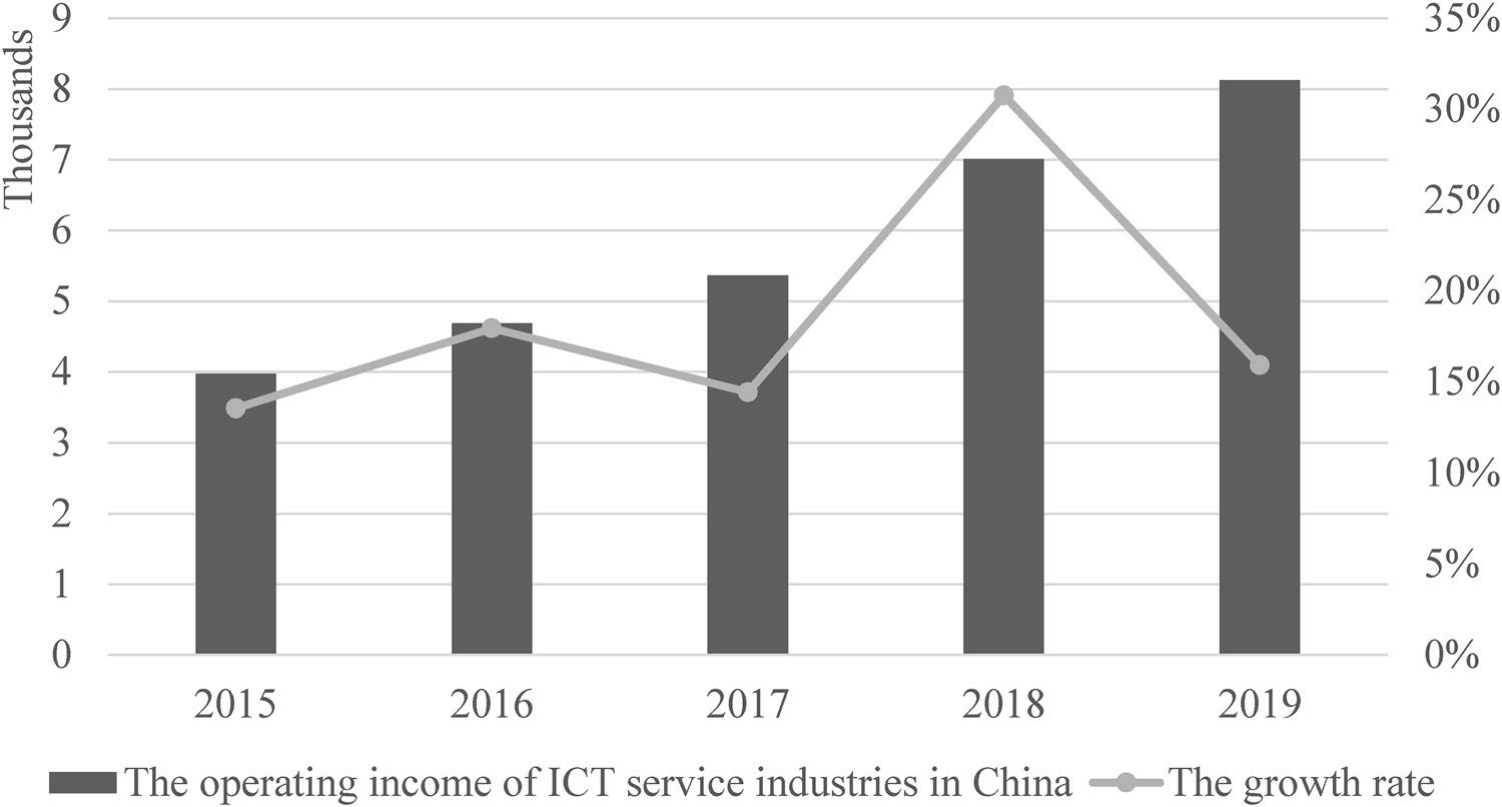
Holistic changes in China’s ICT service industry from 2015 to 2019 (Source: The authors’ edition based on China Statistical Yearbook of the Tertiary Industry, 2016–2020).

Since the start of the twenty-first century, China's ICT has been rapidly developing^26^. Internally, the speed of information infrastructure construction in China is astonishing. Externally, China has unique advantages in mobile payment, e-commerce, and other “Internet plus” strategic practices. Compared with the ICT manufacturing industry, which heavily relies on importing most of its key components and technologies from other countries (such as computer chips, high-performance video cards, and advanced lithography machines), the ICT service industry shows a much more robust growth momentum in China, particularly in the past few years. It is worth noting that, in 2015, their added value exceeded that of ICT manufacturing industries^27^. In addition, China's ICT industry is gradually transforming from equipment manufacturing to high-tech services and has become one of the burgeoning industries at a high and stable speed (Fig. 3). Furthermore, cyberspace constructed by the ICT service industry is increasingly and closely linked with physical space^28,29^. However, the intervention of virtual space aggravates the imbalance of space development. Its rapid development raises a new question: does this rapid development aggravate the imbalance of spatial development in poor areas with poor economic and infrastructure levels?

Therefore, this research focuses on characteristics of spatio-temporal evolution and influencing factors in the ICT service industry at the provincial level in China. This spatial level is important in a country like China, where regional development and industrial development are strongly correlated. This study first analyses the spatio-temporal changes in the ICT service industry in China's 31 provinces (including autonomous regions and municipalities) from 2015 to 2019 by the location quotient (LQ). In the second step, the global and local spatial autocorrelation are applied to analyse the spatial correlation of the industries during this period in China. The global spatial autocorrelation is measured by Moran's I coefficient, reflecting the overall trend of the spatial correlation in China. Meanwhile, the local spatial autocorrelation shows the type and degree of spatial correlation at the province level and surrounding areas, represented by a LISA map. Thirdly, spatial econometric models are utilised to analyse the influencing factors of the ICT service industry. This research can help formulate scientific and reasonable development policies for the ICT service industry. This study's findings can also provide a scientific basis and theoretical support for the industries' distribution of strategies and resources.

## Literature review

Several existing studies have verified numerous effects of the ICT industry on socio-economic development. The first one is that they can improve communication and governance. ICT can create inclusion, efficiency, and innovation^26^. The use of the Internet makes trade easier, resulting in a cost-effective process in everyday operations^30^. By reducing trade costs, such as transport costs and market entry costs, the ICT industry provides new channels of communication to generate new or improved transaction incentives^31,32^. Moreover, they are seen as a driver of government transformation, with local governments playing more of a facilitator role^33^. Secondly, the ICT industry has changed the spatial structure of regions. Cyberspace constructed by industries is increasingly linked more closely with the physical space^28^. However, the intervention of virtual space aggravates the imbalance of space development. For example, Beaverstock, Smith^29^ proposed that ICT promotes the change of Meta-geography in that regions exist in the connection and network rather than the mosaic constituted by one country. Thirdly, most of the empirical studies considering the relationship between the ICT industry and economic growth have concluded that a positive and significant relationship exists^34^. This industry has a wide array of effects on key global economic systems^35^. The rapid use and expansion of these technologies have several economic consequences ranging from increasing productivity and boosting economic growth to reducing corruption^36,37^. Fernandez-Portillo, Almodovar-Gonzalez^38^ concluded that investing in the deployment of ICT supports the sustainable economic development of European Union countries. Thus, the ICT industry increases economic growth in both rich and poor nations. More importantly, poorer nations tend to benefit more than rich countries from the ICT revolution^39^.

Based on the steadily weakening position of manufacturing in global value chains^40^, the ICT service industry shows a significant role in development, compared with the ICT manufacturing industry. This industry is both outputs from the ICT industry and inputs into the ICT-using industry^41,42^. This can provide technical solutions for green and sustainable development^43,44^. For example, Mourshed, Robert^45^ argue that its development is an important factor in future smart grid construction. Digital operations and regional power integration can improve industrial energy efficiency, achieving energy saving, emission decrease, and environmental protection effects^46^. In terms of global practices, the ICT service industry reflects a broader trend of specialisation. As a “glue” in global value chains^47^, the ICT service industry contributes substantially to gross domestic product^48^ and knowledge-intensive employment^49^. More scholars believe that its use can decrease carbon dioxide emissions and promote energy efficiency to a certain extent in the “Belt and Road” countries^50–52^. The Italian government has built an energy network plan based on information and communication technology development and proposed an energy network plan that integrates smart energy storage and on-demand power dispatch^53^. Based on seven projects in the United States (US), Germany, and Italy^54^, indicate that ICT services can reduce energy vulnerability by promoting renewable energy development. Furthermore, this industry has a positive effect on the participation of the Central and Eastern European economies in global value chains^40,55^. It can drive the development of peripheral areas through innovation cooperation^56,57^. Its adoption into firms and public service institutions is an innovation due to strongly enhanced productivity and overall efficiency^42^. As such, there is a need to further explore the ICT service industry to improve its specialisation in different fields, such as its spatio-temporal distribution and evolution.

Spatio-temporal distribution and the evolution of the ICT service industry are already clarified and developed in the literature. For example, Jøranli and Herstad^58^ investigated the urban concentration of ICT service employment in Norway from the perspective of labour market linkages and implied that the industry would continue to concentrate in the large-city regions. The main reason is that employment in the services segment of the ICT industry itself has exhibited exceptionally high levels of concentration in large, high-cost, and often congested urban locations^59^. This concentration is resilient, despite the growing importance of global innovation networks^60^ and the current trend of service export and outsourcing to low-cost countries, such as India^61^. Additionally, African firms that use the ICT service industry increases productivity compared to non-users^62^. This does not necessarily imply that just having ICT service is a guarantee to have better economic growth. In the adoption and successful utilisation of ICT in resource-rich countries, institutions, cultural factors, and mindsets are key players. While countries such as Saudi Arabia and Qatar possess good ICT infrastructure, the lack of suitable institutions that complement human capital hinders the transformation of ICT into productive activities^42^. As seen, existing studies have analysed the effects and characteristics of the ICT service industry at the national level. Based on the role of spatial proximity between regions in the location selection of service industries^63^, a few studies, however, have concentrated on the spatial and temporal differences in the ICT service industry at the regional scale.

As the largest developing country with limited sources, any burgeoning industries in China should pay attention to their distribution based on their spatio-temporal differences^22,64,65^. The distribution and evolution of many industries in China have been fully verified, such as the shipping service industry^66^, smart industries^67^, and green industries^68^. It is shown that these industries in the east are more well-developed than those in the west. In the context of the increasing spatial correlation of China’s service industry specialisation, it is unrealistic to study the development characteristics of the ICT service industry without spatial correlation. Therefore, research on the spatio-temporal distribution and evolution of the ICT service industry at the sub-national level is essential, while this kind of study for China remains absent. Provincial space is the most basic regional unit of socio-economic development in China. It provides institutional governance and guidance while leading the main direction and distribution of any type of industrial development within the regions. We can argue that the provincial level is of the most interest to this type of research in China. Thus, this study endeavours to fill these gaps. The next section provides a brief outline of materials and methods to address those research objectives.

## Research methods and data

To explain and reveal the characteristics and laws of the ICT service industry on the macro scale, the spatial comparison of the ICT service industry in China's provincial revenue distribution is carried out. This study adopts the spatial econometric analysis methods of location entropy analysis and spatial autocorrelation analysis to measure and analyse the degree of industrial spatial specialisation and adjacent characteristics, respectively. It then summarises the spatial pattern and spatial relationship of China's ICT service industry.

### Location quotient

This study uses provincial attribute data to show the spatial differentiation characteristics of China's ICT service industry. The location quotient (LQ) is often used to measure the level and degree of specialisation of a particular industry in a certain region. In other words, LQ is used to describe the relative ratio of the ICT service industry in a province to that in a country. The operating income of the ICT service industry was selected as the data source for the study of spatial differentiation of the industry in China. The equation is:

1 $$\begin{array}{*{20}c} {LQ = \frac{{l_{i} /\mathop \sum \nolimits_{j = 1}^{m} l_{i} }}{{L_{i} /\mathop \sum \nolimits_{j = 1}^{m} L_{i} }}} \\ \end{array}$$

where ${l}_{i}$ is the operating income of the ICT service industry of a province, ${L}_{i}$ is the value-added of tertiary industry, and $m$ represents the number of regions.

If LQ > 1, the specialisation level of this industry in the province is higher than the national average level, which has a strong competitive advantage and can timely increase the strength of the external expansion. If LQ = 1, it indicates that the level is equal to the national average one that the industrial specialisation phenomenon is not obvious. If LQ < 1, the level is lower than the national average one, which indicates no obvious specialisation. This industry needs to be imported from outside the region to meet the needs of production and living in the province. In other words, the larger LQ means that the ICT service industry in the province usually forms industrial clusters in space. On the contrary, the spatial distribution is more dispersed.

### Spatial autocorrelation analysis

Spatial autocorrelation is the degree to which a geographic phenomenon of a regional unit or the value of an attribute is related to the same phenomenon or attribute value on an adjacent unit^69^. The global and local spatial autocorrelation are applied to analyse spatial data for spatial correlation analysis of data in the research area. Spatial autocorrelation is divided into global spatial autocorrelation and local spatial autocorrelation^70^. The global measurement mainly includes Moran’s I statistic, and the local spatial autocorrelation is measured by the Local Moran’s I index^71^. Moran's I coefficient is used to measure global spatial autocorrelation, which reflects the overall characteristics and trend of spatial correlation of the ICT service industry in the whole research area. The following equation is used to determine the global spatial autocorrelation:

2 $$\begin{array}{*{20}c} {Moran^{\prime}s I = \frac{{m\mathop \sum \nolimits_{i = 1}^{m} \mathop \sum \nolimits_{j = 1}^{m} w_{ij} \left( {x_{i} - \overline{x}} \right)\left( {x_{j} - \overline{x}} \right)}}{{\mathop \sum \nolimits_{i = 1}^{m} \mathop \sum \nolimits_{j = 1}^{m} w_{ij} \mathop \sum \nolimits_{i = 1}^{m} \left( {x_{i} - \overline{x}} \right)^{2} }}} \\ \end{array}$$

where $Mora{n}^{^{\prime}}s I$ is the global spatial autocorrelation index. ${x}_{i}$ and ${x}_{j}$ are the index values of samples $i$ and $j$, respectively. $\overline{x }$ refers to the average value of the sample index and ${w}_{ij}$ is the spatial relationship weight matrix. $Mora{n}^{^{\prime}}s I$ is between -1 and 1.

If $I$>0, the global spatio-temporal autocorrelation of observations is positive and takes on that with the adjacency of high-high values or low-low values in both time and space, and the higher value presents a stronger positive relationship. On the contrary, if $I$<0, the autocorrelation is negative and presents a spatio-temporal scattered pattern with high-low values, and the lower $I$ is, the stronger the negative relationship. Additionally, if $I$=0, the observations are distributed independently and randomly. Furthermore, the z-score and p-value are used to evaluate the significance of this index.

The global index $Mora{n}^{^{\prime}}s I$ is a synthesised measurement index for the overall spatio-temporal autocorrelation, disclosing the spatio-temporal association pattern of the whole study area. Nevertheless, it neglects the potential instability of the spatio-temporal process and local spatial heterogeneity, reflecting the difference between different spatio-temporal objects in the region^72^. Hence, the local index should be applied to reflect further the heterogeneous properties of this difference to reveal the local spatio-temporal association structure. In this paper, local spatial autocorrelation is used to analyse the spatial characteristics and degrees of each province and the surrounding provinces. These are generally represented by local indicators of spatial association (LISA) diagrams. The following equation is used to determine the local spatial autocorrelation:

3 $$\begin{array}{c}{I}_{i}=\frac{m\left({x}_{i}-\overline{x }\right)\sum_{j=1}^{m}{w}_{ij}\left({x}_{j}-\overline{x }\right)}{\sum_{j=1}^{m}{\left({x}_{j}-\overline{x }\right)}^{2}}\end{array}$$

Similar to global spatial autocorrelation, the local spatio-temporal association can also be divided into four types: high-high (HH), low-low (LL), high-low (HL), and low–high (LH) values. If ${I}_{i}$ is positive, it indicates that the local area is a LL or HH clustering area. A local positive spatio-temporal autocorrelation exists between a province and its adjacent provinces with a spatio-temporal conglomeration effect of the same high or low values. Negative ${I}_{i}$ indicates that the local area is a LH or HL clustering area. Local negative spatio-temporal autocorrelation, of which the high and low different values are adjacent, reflects that the observations of adjacent provinces in the two types of local spatio-temporal association are of great disparity with each other.

### Spatial econometric model

China’s ICT service industry may be spatially correlated, which cannot meet the assumption of regional independence in the traditional linear regression model. The spatial econometric model is therefore taken into consideration to analyse the influencing factors of the ICT service industry. In order to adapt the conventional OLS regression model, the spatial weight matrix is added. Furthermore, the spatial panel and cross-section econometric models are both parts of the spatial econometric model. The spatial panel econometric models are used for this study based on the kind of dataset. Among the spatial panel econometric models are the Spatial Lag Model (SLM), and the Spatial Error Model (SEM). SEM is used when the model's error term exhibits spatial correlation, while SLM is used when the explained variables exhibit strong spatial correlation. Moreover, the LM test, LR test and Wald test proposed by^73^ are adopted to assess and select the spatial econometric models. The full spatial econometric model with all types of interaction effects of the dependent and independent variables takes the following form:

4 $$\begin{array}{c}{y}_{it}=\delta \sum_{j=1}^{N}{W}_{ij}{y}_{jt}+\alpha \iota N+\sum_{j=1}^{N}{\mathrm{W}}_{ij}{x}_{ijt}\theta +uu=\rho \sum_{j=1}^{N}{W}_{ij}{u}_{jt}+\varepsilon \end{array}$$

where $\delta$ represents the spatial autoregressive coefficient, θ denotes a K × 1 vector of fixed but unknown parameters to be estimated and $\rho$ is the spatial autocorrelation coefficient. $W$ is a nonnegative i × j matrix describing the spatial configuration or arrangement of the units in the sample. ${W}_{ij}{y}_{jt}$ is the endogenous interaction effects among the dependent variable of the different provinces. ${W}_{ij}{x}_{ijt}$ is the exogenous interaction effects among the independent variables. ${W}_{ij}{y}_{jt}$ denotes the interaction effects among the disturbance term of the different units^74,75^.

### Definition of variables

Based on the definition of China’s ICT service industry and its prior global studies^42,56–58,67^, eleven variables are selected as the potential influencing factors of China’s ICT service industry: fixed telephone user (FTU), mobile phone user (MPU), length of long-distance optical cable (LLOC), telephone universality rate (TUR), number of domain names (NDN), number of websites (NW), mobile internet access traffic (MIAT), population, GDP, area and ICT employment. Two variables, MPU and population have significant multicollinearity with other explanatory variables, thus being excluded from the analysis. These fileted variables and their description is shown in Table 1.

**Table 1:** The description of the variables.

Abbreviation	Variables	Description
FTU	Fixed Telephone User	All telephone users who have gone through account registration procedures at the business outlets of telecom enterprises and have connected to the fixed telephone network
LLOC	Length of Long-distance Optical Cable	Optical cable assembly that acts as a transmission medium and can be used individually or in groups
TUR	Telephone Universality Rate	Total number of telephones (including mobile phones)/ Total Population of administrative Area
DN	Domain Number	It provides easily recognisable and memorable names to numerically addressed Internet resources
WPN	Web Page Number	It can monitor the operation of the websites
MIAT	Mobile Internet Access Traffic	Total data traffic of various mobile networks (excluding SMS and MMS)
GDP	GDP	Those that are bought by the final user-produced in a country in a given period
Area	Area	The administrative area of land
ICT Employment	ICT Employment	The population working in ICT services and receiving remuneration or business income

Before the formal regression analysis, Table 2 shows the simple correlation between any two variables in the econometric model, which helps offer some preliminary insights. The variables FTU, TUR, DN, WPN and MIAT have positive correlations with the ICT service industry. These factors can reflect and drive the development of the industry. Also, the two variables, GDP and ICT employment, are significantly positively correlated with this industry, suggesting the development of ICT services could be better in those labour-intensive and economically fast-developed provinces. Although the correlation analysis offers us some preliminary insights, they are not rigorous to indicate valid causal inferences, such that we still need to rely on rigorous regression analysis.

**Table 2:** Correlation test of variables.

	ICT service industry	FTU	LLOC	TUR	DN	WPN	MIAT	GDP	Area	ICT employment
ICT service industry	1	0.593**	−0.124	0.836**	0.644**	0.902**	0.546**	0.646**	−0.292	0.959**
FTU	0.593**	1	0.425*	0.351	0.600**	0.312	0.862**	0.874**	−0.223	0.571**
LLOC	−0.124	0.425*	1	−0.274	0.032	−0.214	0.340	0.208	0.486**	−0.072
TUR	0.836**	0.351	−0.274	1	0.408*	0.782**	0.160	0.302	−0.224	0.787**
NDN	0.644**	0.600**	0.032	0.408*	1	0.577**	0.602**	0.665**	−0.352	0.673**
NW	0.902**	0.312	−0.214	0.782**	0.577**	1	0.287	0.367*	−0.256	0.900**
MIAT	0.546**	0.862**	0.340	0.160	0.602**	0.287	1	0.913**	−0.268	0.510**
GDP	0.646**	0.874**	0.208	0.302	0.665**	0.367*	0.913**	1	−0.322	0.620**
Area	−0.292	−0.223	0.486**	−0.224	−0.352	−0.256	−0.268	−0.322	1	−0.298
ICT employment	0.959**	0.571**	−0.072	0.787**	0.673**	0.900**	0.510**	0.620**	−0.298	1

** And * indicate significant levels of 1% and 5%, respectively.

### Data source

This research selects 31 provincial administrative districts in China. Taiwan, Hong Kong, and Macao were excluded due to data limitations and the fact they do not fit the research area and scale of the study. The statistical scope of the ICT service industry in this research referred to the classification defined above, including telecommunications, computer programming, consultancy and related activities, data processing, hosting and related activities, web portals, and repair of computers and communication equipment. Generally, their levels were digitised as their operating income in China. Moreover, as mentioned earlier, their added value exceeded that of the corresponding manufacturing in 2015. Therefore, the research period is from 2015 to 2019, focusing on three nodes in 2015, 2017 and 2019. The data includes the ICT service industry's operating income and the tertiary industry's value-added, which are collected from the China Statistical Yearbook of the Tertiary Industry (2016–2020), published by the National Statistical Department. Additionally, the statistical data of GDP, area and ICT employment derives from China Statistical Yearbook 2020. Other relevant data is from the China Statistical Yearbook of the Tertiary Industry 2020. Table 3 shows descriptive statistics of the variables involved in this research. Aligned with this study’s objectives, these data are further analysed.

**Table 3:** Descriptive statistics of the variables.

	Unit	Mean	St.d	Min	Max
FTU	Persons	616.235	509.8126	53.9	2303.3
LLOC	Km	34,997.94	23,424.912	3215	122,353
TUR	%	129.2658	25.62021	98.90	211.87
NDN	PCS	161.535	168.5907	2.0	695.1
NW	PCS	92,233.7	9223.4	382.8	11,249,165.1
MIAT	10,000 GB	92,233.7	9223.4	34,381.3	1,379,371.0
GDP	100,000,000 RMB	31,784.9	9223.4	1697.8	107,671.1
Area	km^2^	31.0781	38.77182	0.63	166.49
ICT employment	Persons	14.685735	19.0312639	0.7947	85.9131

## Results

The results of the analyses were mainly divided into two parts, including characteristics of the holistic and local spatio-temporal evolution of the ICT service industry. A detailed explanation of holistic temporal change characteristics of this industry from 2015 to 2019 in China is essential as it can reflect the upper level of trend and the whole situation. However, they were roughly defined in the introduction section. This section analyses spatial evolution characteristics, including the differentiation and correlation of the industry among those provinces, not only accessing the space changes but based on the progression of time.

### Spatial differentiation characteristics of the ICT service industry

Table 4 provides statistics on the specialisation degree and the change rate of the ICT service industry in each province from 2015 to 2019. The degree in ten provinces increased during the sample period. For example, the degree in Xinjiang (LQ = 0.52 in 2015 and 0.88 in 2019) had a disadvantage at the beginning of the period and turned into a dominant province at the end. In addition, the LQs of the ICT service industry in Beijing (3.53/3.41/3.52), Shanghai (1.86/1.74/1.85), Zhejiang (1.53/1.96/1.88), Tibet (1.42/1.76/1.65) and Guangdong (1.14/1.23/1.41) in 2015, 2017, and 2019 were greater than 1 point. Among them, Beijing's LQ for those three years was all over 3 points. In other words, China's ICT service industry during the period was mainly concentrated in these five provinces, implying that ICT service in China is mainly dependent on economically developed regions. Especially, Tibet was economically backward, but its LQ was high. The underlying reasons are manifold and will be explored in the next section. Although some provinces maintained a high level of industrial development, other provinces have low levels. According to the 21 provinces with declining specialisation degrees, the degrees in western provinces (except for Tibet and Xinjiang) decreased significantly, and Yunnan and Sichuan experienced a drop of more than 40%. Moreover, although the degrees in Heilongjiang, Henan, and Hubei had improved during the sample period, they were still inferior provinces at the end of the period.

**Table 4:** Specialisation and its changes in different provinces (Source: The authors’ edition using the statistical reports).

	Province	LQ in 2015	LQ in 2019	Change (%)
Provinces with increasing specialisation	Xinjiang	0.52	0.88	68.91
	Tianjin	0.73	0.98	34.59
	Heilongjiang	0.36	0.44	24.41
	Guangdong	1.14	1.41	23.15
	Zhejiang	1.53	1.88	22.93
	Tibet	1.42	1.65	16.28
	Henan	0.47	0.54	14.14
	Hainan	0.79	0.86	9.71
	Hubei	0.55	0.59	5.92
	Liaoning	0.52	0.55	5.89
Provinces with decreasing specialisation	Beijing	3.53	3.52	−0.37
	Shanghai	1.86	1.85	−0.37
	Fujian	0.75	0.72	−3.54
	Hebei	0.52	0.49	−5.47
	Shandong	0.58	0.53	−8.8
	Jiangxi	0.59	0.52	−10.92
	Hunan	0.47	0.41	−13
	Shaanxi	1.01	0.87	−14.23
	Guangxi	0.5	0.43	−15.36
	Jiangsu	1.07	0.85	−20.9
	Gansu	0.58	0.45	−22.23
	Jilin	0.6	0.46	−23.07
	Shanxi	0.42	0.32	−23.93
	Anhui	0.66	0.5	−24.35
	Guizhou	0.68	0.49	−28.02
	Inner Mongolia	0.38	0.27	−28.17
	Chongqing	0.87	0.61	−29.45
	Ningxia	0.57	0.39	−31.63
	Qinghai	0.65	0.44	−32.7
	Sichuan	1.18	0.67	−43.15
	Yunnan	0.71	0.4	−44.53

**Figure 4:**
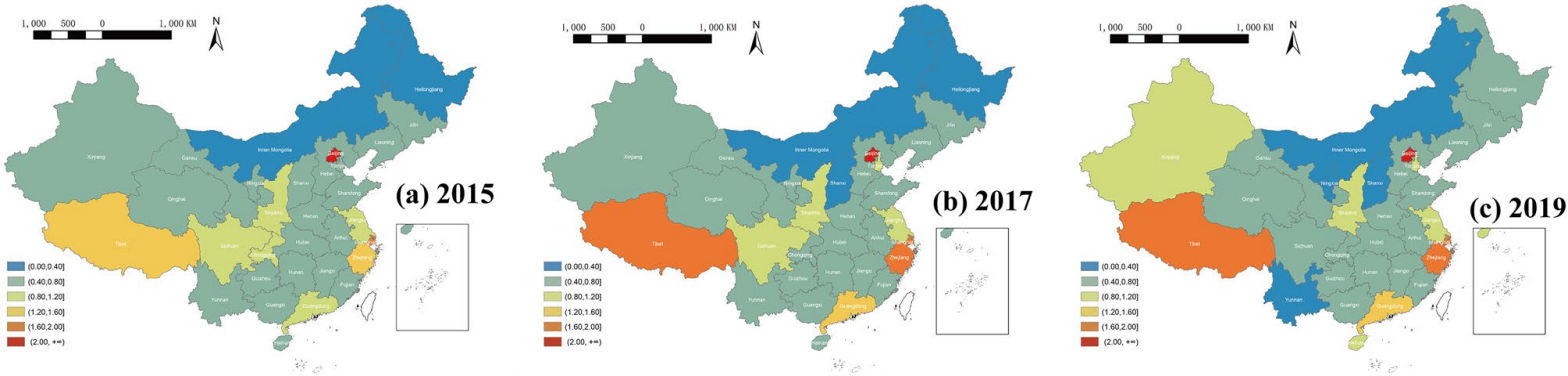
Spatial distribution of the ICT service industry in China, from 2015 to 2019 (Source: The authors’ own, using available data from 2015 (**a**), 2017 (**b**), and 2019 (**c**), made in ArcGIS 10.8, https://www.esri.com/zh-cn/arcgis/products/arcgis-desktop/resources).

In terms of spatial distribution, the ICT service industry shows a trend of specialisation development. Meanwhile, some polar cores gradually strengthen their specialisation tendency, being in the early stage of multi-core development^67^. The industry had initially formed a cluster development trend (see Fig. 4). During the sample period, the five provinces mentioned above served as development cores, driving the development of surrounding provinces, such as Xinjiang, Hainan, and Tianjin, indicating that sub-core regions of ICT services were emerging. Nevertheless, the development gap between regions still existed. This industry in other provinces was still in the stage of dispersion and slow rise without a cluster point. In particular, in the northern provinces of Inner Mongolia, Gansu, and Shanxi, the ICT services were gradually being replaced by other industries.

### Spatial correlation characteristics of the ICT service industry

To estimate the spatial agglomeration and evolution characteristics of the ICT service industry, the global autocorrelation model was used to measure Moran’s I (Table 5). The distance ${D}_{ij}$ between provinces used to calculate the weight coefficients ${w}_{ij}$ in the $Mora{n}^{^{\prime}}s I$, was created by the matric of Euclidean Distance with the automatic minimum 10.162089 bandwidth. The spatial correlation of this industry from 2015 to 2019 was positive with *p* < 0.05, indicating that this was significant at a 5% of confidence level. The values of Moran’s I was greater than zero, indicating that the provinces had a positive spatial agglomeration effect on the ICT service industry.

**Table 5:** Global Moran's I test (Source: The authors’ own).

Year	*I*	*E(I)*	*SD(I)*	*z*	*p*
2015	0.125	−0.033	0.006	2.115	0.034
2017	0.120	−0.033	0.006	2.053	0.040
2019	0.133	−0.033	0.006	2.219	0.027

**Figure 5:**
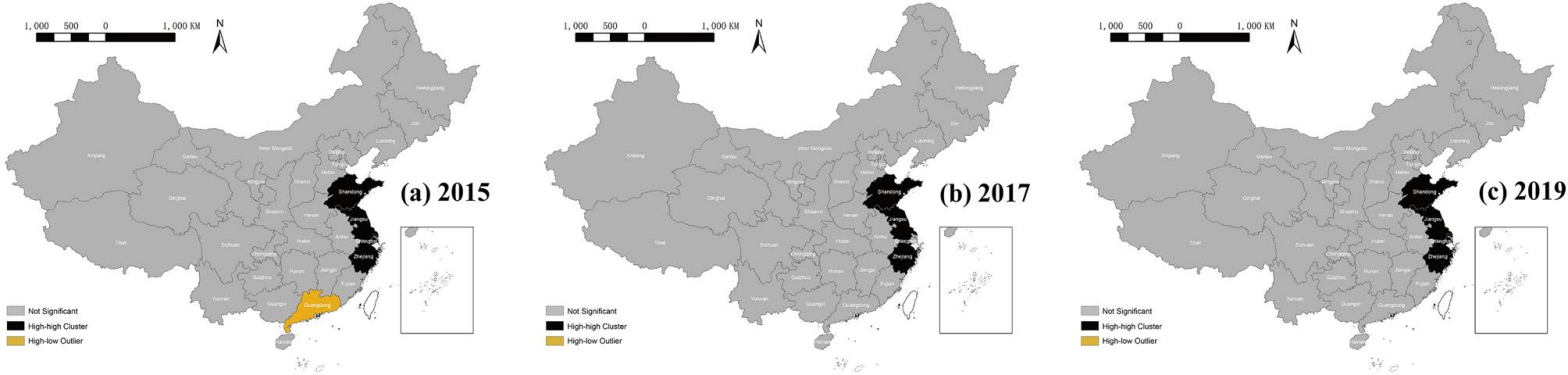
LISA diagrams of the ICT service industry in China in (**a**) 2015, (**b**) 2017, and (**c**) 2019 (Source: The authors’ own, made in ArcGIS 10.8, https://www.esri.com/zh-cn/arcgis/products/arcgis-desktop/resources).

The local LISA diagrams of the ICT service industry in 2015, 2017, and 2019 showed three main characteristics (see Fig. 5). Firstly, numbers and types of local spatio-temporal association were kept stable in the period. 5, 3, and 4 provinces showed significance in 2015, 2017, and 2019, respectively. The rest did not pass the significance test, indicating that the existing data could not identify these as association types. Additionally, only the high-high (HH) type existed in this period, except Guangdong showed the high-low (HL) type in 2015. Secondly, their distribution difference was noticeable, reflecting that the local spatio-temporal heterogeneity areas varied significantly. The four provinces in eastern coastal areas, including Zhejiang, Shanghai, Jiang, and Shandong, were located in the HH clusters with the exception that Shanghai did not show this characteristic in 2017, but this re-emerged in 2019, reflecting that the development levels of this industry in these areas are relatively high, radiating and driving the surrounding cities. It also verifies the spatial relation of global space positive autocorrelation, as mentioned above. Lastly, the development of the ICT service industry in some areas showed a significant imbalance. Guangdong was in the HL area in 2015, indicating that the development level of its industry was relatively high-value, while that in surrounding provinces, including Guangxi, Hunan, Jiangxi, and Hainan, was relatively low. This situation, however, did not exist after 2015. The industry development became balanced among those provinces. Overall, the spatial pattern of China's ICT service industry did not change significantly.

### Influencing factors of the ICT service industry

The above spatial correlation analysis shows that OLS is not suitable for exploring the influencing factors of China’s ICT service industry, while the spatial econometric models are more appropriate. According to the test ideas proposed by Elhorst^73^, the Lagrange Multiplier (LM) test is used to select the spatial econometric model. Table 6 shows the results of the test. LM-Lag and robust LM-Lag test reject the hypothesis that the explained variables do not exist in spatial autocorrelation, so the spatial lag model (SLM) should be used. LM-Error and robust LM-Error tests do not reject the null hypothesis that random errors have spatial autocorrelation, so the spatial error model (SEM) should not be used. Furthermore, the coefficient $\rho$ of the SLM is significant at the 5% level, and the coefficient $\lambda$ of the SEM is significant at the 10% level, indicating that the spatial correlation of this industry is more affected by the explanatory variables of adjacent spatial units than by the perturbation error term. If the spatial Durbin model (SDM) based on adjacent space perturbation error is used, there may be a large error in the results. Therefore, the SLM is constructed to discuss the influencing factors of China’s ICT service industry.

**Table 6:** The results of LM tests.

Test	MI/DF	Value	Probability
LM-Lag	1	4.8793	0.02718
Robust LM-Lag	1	4.1919	0.04062
LM-Error	1	3.1822	0.07445
Robust LM- Error	1	2.4948	0.11422

The regression results of the SLM are shown in Table 7. The R^2^ in Table 7 is 0.9928, indicating that the explanatory variables we selected explain 99.28% of the ICT service industry, which is statistically acceptable. Another 0.72% may include unobserved socio-economic or natural factors. The following is an interpretation of the regression results.

**Table 7:** The regression results of the SLM.

Variables	Coefficient
FTU	0.0035 (0.1280)
LLOC	−0.0029 (0.8175)
TUR	0.0009** (0.0300)
NDN	0.2613** (0.0154)
NW	−0.2821*** (0.0000)
MIAT	0.0001*** (0.0000)
GDP	−0.0001*** (0.0000)
Area	0.0066*** (0.0000)
ICT Employment	−0.5402** (0.0102)
N	31
R-square	0.9928

1. The values in parenthesis are P values of the estimated coefficients.

2. ***, ** and * indicate significant levels of 1%, 5% and 10%, respectively.

The coefficient of FTU is positive, and it has passed the significance test of 5%, which shows that the higher the popularity of the telephone is, the higher level of the ICT service industry will be. As the traditional infrastructure of ICT service, the telephone has been well developed. Its industrial development plays a significant role in promoting the basic platform. The coefficient of NDN is positive and significant at 5%, which shows that the increase in domain names has brought advanced internet technology and technology spillover effects through basic information exchanges. NDN reflects the level of internet development in a region. Its promotion means the further development of the internet industry and then improves of the ICT service industry. The coefficient of NW is negative and significant at 1%, which shows that the fewer websites are browsed in a province, the higher level of its ICT service industry will be. The increase in NW may lead to more complex information retrieval and pay more attention to enjoying the outcomes of ICT service techniques from other provinces or countries rather than developing them, resulting in a low level of ICT service industry. The coefficient of MIAT is positive and significant at the level of 1%, which shows that the more mobile internet access traffic is used in a province, the higher level of its ICT service industry will be. MIAT can reflect the usage amount of ICT services at a specific time. More traffic brought more online communication. The coefficient of GDP is negative and significant at 1%, which shows that the better the economic development of the province, the worse the industrial development of ICT services. Less attention is likely to be paid to ICT services as resources are channelled into other industries to promote economic development. The coefficient of the area is positive and significant at 1%, which shows that ICT services are likely to develop better in larger provinces. Although the ICT service industry transcends geographical, spatial and administrative boundaries, industrial agglomeration effect and spatial correlation brought by area still exist significantly. The coefficient of ICT employment is negative and significant at the level of 5%, indicating that the on-site employees may drag on the development of the ICT service industry probably because most of them can remotely work online without the expenditure for their companies. Oppositely, the coefficients of FTU and LLOC are not significant.

## Discussion and conclusions

The ICT service industry has become an important pillar in China's industrial development. Their equitable distribution can improve the levels of national and global positive peace. The spatio-temporal autocorrelation methods, which attend to both spatial and temporal effects simultaneously, and spatial econometric models, were introduced in this paper to explore the characteristics of spatio-temporal evolution in the ICT service industry at a sub-national scale and its influencing factors in China. The results showed that the ICT service industry in China during the period between 2015 and 2019 was not only distributed and concentrated in cities with relatively superior overall development levels but also those cities with superior industrial and development carrier elements. It was found that China's ICT service industry is mainly concentrated in Beijing, Shanghai, Zhejiang, Tibet, and Guangdong with a trend of specialisation development. On the one hand, in provinces with high economic development, such as Shanghai, Beijing, and Guangzhou, the share and growth of this industry showed a positive trend from 2015 to 2019. As Jøranli and Herstad^58^ presumed before, these were concentrated in large, high-cost, and congested regions, due to technology intersection and industry convergence, presenting the characteristics of path dependence^59^. In other words, technological relevance and aggregation play a significant role in promoting the emergence and development of this industry. On the other hand, economically underdeveloped regions supported by the system, such as Xinjiang and Tibet, also showed high proportions and growth. It was different from some other industries in the east that are more well-developed than those in the west of China^67^. The main reason might be that the development of the ICT service industry was rooted in a regional policy system, which could be an incentive or an obstacle to the evolution of regional industries. The formulation and implementation of preferential policies would directly affect the development and agglomeration of these industries^33^. For example, by building a new computing power network system integrated with data centres, cloud computing, and big data, the east's computing power needs would be guided to the west in an orderly manner, and the layout of data centre construction would be optimized to promote the coordination between the east and the west.

Furthermore, the global and local spatial autocorrelation were applied to analyse the spatial correlation of this industry during the period in China. The ICT service industry was characterised by stable and highly concentrated development. Based on the result of the global autocorrelation, factor flow barriers between administrative regions were further reduced, and the correlation of this industry between regions was enhanced. Its development in a certain region had a significant spatial spillover effect on the development of adjacent areas, resulting in spatial agglomeration and multi-core development^67^. The positive spatial correlation of this industry was mainly concentrated in four eastern coastal provinces: Zhejiang, Shanghai, Jiangsu, and Shandong, which was consistent with the characteristics of the ICT industry that Cyberspace constructed by this industry was increasingly closely linked with physical space^28^. Unlike the ICT research^29^, the ICT service industry never aggravated the imbalance of space development. In addition, geographical proximity was also an important driving force for its development. The result of local autocorrelation showed a definite spatial correlation in spatial distribution with constant strengthening. Areas with high-level development of the ICT service industry drove the development of neighbouring regions to a certain extent. For example, the HL characteristic in Guangdong in 2015 disappeared in 2017 and 2019. Additionally, TUR, NDN, MIAT and the area were shown to have a significant role in promoting the ICT service industry, while NW, GDP and ICT Employment were shown to have a significant negative impact on this industry. The outcomes can improve resource integration from the national perspective, optimise resource allocation, and improve resource use efficiency.

Two improvement strategies are put forward to correspond to the above characteristics of spatio-temporal evolution in the ICT service industry in China. The first is to accelerate the inter-provincial networking development of the ICT service industry. Industrial networking is the key node of China's economic growth at present. As an effective means to promote industrial agglomeration, it plays an irreplaceable role in policy platform support, large data, logistics platform, and other high-quality coordination of infrastructure. Moreover, it acts as a window demonstration role in the core area and radiates and drives the industrial development of surrounding regions. The second is to strengthen government policy guidance for the ICT service industry. It is in a period of rapid development and transformation, and the policies, institutions, and governance mechanisms implemented by the central and local governments affect the whole process of industrial development. Relevant policymakers should take the initiative to adjust the institutional framework and establish a hierarchical policy system from top to bottom. Differentiated policies considering local characteristics should be developed based on regional advantages, and the synergy of development power inside and outside the ICT service industry will be realised by emphasising the interaction between market mechanisms and government policy.

Finally, there are some limitations and deficiencies in this research. This mainly focused on the spatial agglomeration and location selection of the ICT service industry and paid insufficient attention to the internal industrial organisation and connection. Future research can be further advanced toward the network construction of the ICT service industry, promoting the development of these industries to break through space constraints and avoid geographical lock-in homogeneity. Moreover, certain urban agglomerations should also be explored in future work, such as the Yangtze River Delta, Pearl River Delta, and Beijing-Tianjin-Hebei. The ICT service industry in these urban agglomerations has a clearer division of labour and is closely connected; therefore, the evolution characteristics could be further explored. Other future research directions may include the topics such as sub-national spatio-temporal distribution and e-commerce development, interactions between the ICT service industry and other positive peace pillars, in-depth analysis at city levels, etc.

## Supplementary Information

Supplementary Information.

## Data Availability

The datasets analysed during the current study are available in the China Statistical Yearbooks Database repository, http://www.stats.gov.cn/sj/ndsj.
